# Reviewing the genetics of heterogeneity in depression: operationalizations, manifestations and etiologies

**DOI:** 10.1093/hmg/ddaa115

**Published:** 2020-06-22

**Authors:** Na Cai, Karmel W Choi, Eiko I Fried

**Affiliations:** 1 Helmholtz Pioneer Campus, Helmholtz Zentrum München, Neuherberg 85764, Germany; 2 Department of Psychiatry, Massachusetts General Hospital, Boston, MA 02114, USA; 3 Department of Epidemiology, Harvard T.H. Chan School of Public Health, Boston, MA 02115, USA; 4 Psychiatric and Neurodevelopmental Genetics Unit, Center for Genomic Medicine, Massachusetts General Hospital, Boston, MA 02114, USA; 5 Stanley Center for Psychiatric Research, Broad Institute, Boston, MA 02142, USA; 6 Department of Psychology, Leiden University, Leiden 2333 AK, Netherlands

## Abstract

With progress in genome-wide association studies of depression, from identifying zero hits in ~16 000 individuals in 2013 to 223 hits in more than a million individuals in 2020, understanding the genetic architecture of this debilitating condition no longer appears to be an impossible task. The pressing question now is whether recently discovered variants describe the etiology of a single disease entity. There are a myriad of ways to measure and operationalize depression severity, and major depressive disorder as defined in the Diagnostic and Statistical Manual of Mental Disorders-5 can manifest in more than 10 000 ways based on symptom profiles alone. Variations in developmental timing, comorbidity and environmental contexts across individuals and samples further add to the heterogeneity. With big data increasingly enabling genomic discovery in psychiatry, it is more timely than ever to explicitly disentangle genetic contributions to what is likely ‘depressions’ rather than depression. Here, we introduce three sources of heterogeneity: operationalization, manifestation and etiology. We review recent efforts to identify depression subtypes using clinical and data-driven approaches, examine differences in genetic architecture of depression across contexts, and argue that heterogeneity in operationalizations of depression is likely a considerable source of inconsistency. Finally, we offer recommendations and considerations for the field going forward.

## Introduction

Depression is a common, complex and debilitating condition with a lifetime prevalence of 20% worldwide. Whether it is one unitary construct, or better conceptualized as different and potentially overlapping disorders, has been the subject of vigorous debate over the past decades.

A typical cohort used in genetic studies of depression includes cases with the same diagnosis. However, cases often differ in many respects including symptoms, number of episodes, comorbidities and disease course. This heterogeneity, often hidden and unexamined in genome-wide association studies (GWAS) cohorts, has been identified as one of the main roadblocks to successfully unraveling the genetic architecture of depression, as initial GWAS efforts were limited by both heterogeneity and low power (1,2). In response, many studies relaxed ascertainment criteria to increase sample sizes (3–7), which likely increased rather than decreased heterogeneity within their cohorts. Although this strategy has given us more GWAS associations over the past 5 years, it has also brought into sharper focus the issues of measurement and construct heterogeneity (8).

Heterogeneity is irrelevant if depression reflects a single, specific disorder that carves nature at its joints (9), but work in the last decades should have disabused us of this notion. Instead, depression may consist of various subtypes with different underlying biological pathways and environmental contributions. Systematically studying heterogeneity may be crucial for psychiatric genetics moving forward.

## Sources of heterogeneity

**Figure 1:**
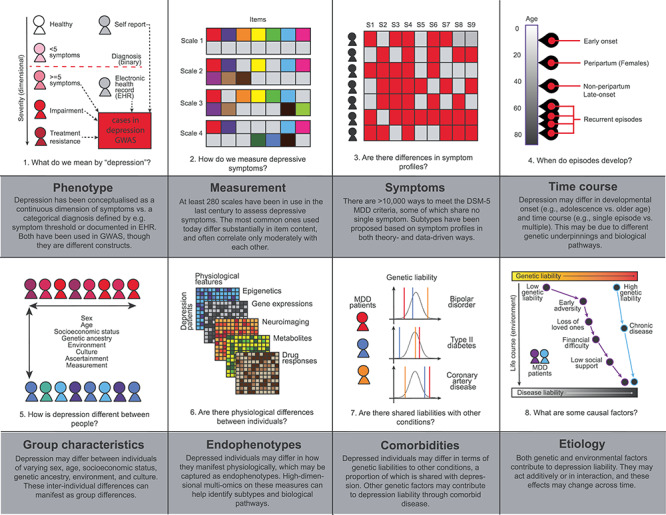
Sources of heterogeneity that impact depression research in terms of operationalization (phenotype, measurement), manifestation (symptoms, time course, group characteristics, endophenotypes, comorbidities), and etiology.

We distinguish three sources of heterogeneity that impact genetic studies of depression, shown in Figure 1.

First, operationalization, including the *construct definition* and its *measurement*. The term ‘depression’ is an umbrella term that has been used to refer to, among others, depressive symptoms as a continuum and clinical depression as a category. The Diagnostic and Statistical Manual of Mental Disorders (DSM-5) (10) offers a formal definition of major depressive disorder (MDD); criteria encompass the presence and duration of key symptoms as well as their cumulative functional impairment. Yet, it was not created with the goal to define a genetically homogeneous phenotype, and have been shaped by the complex history of psychiatry (11,12). Over 280 rating scales have been used to assess depressive symptoms, and common scales only overlap moderately in symptom content (13). Clinical diagnoses and self-report measures have been used to determine depression cases for genetic studies, but rely on different criteria and identify sets of cases that do not fully overlap. Minimal phenotyping approaches may assess a different construct than MDD specified by DSM-5 (8), and referring to all these phenotypes as ‘major depression’ (14) obfuscates important differences. Further, cultural differences across ethnicity and nationality may also contribute to heterogeneity in measurement (15–17). In the remainder of this review, we refer to ‘depression’ as all operationalizations described above, and MDD as defined by formal diagnostic criteria (e.g. DSM-5).

Second, manifestations, which encompass *symptoms*, *severity*, *developmental timing*, *comorbidities and physiology*. DSM-5 criteria for MDD include diverse symptoms such as low mood, loss of interest, sleep disturbance, weight changes, psychomotor slowing/agitation and suicidal ideation. There are up to 10 377 unique ways to meet these diagnostic criteria (18), and cohorts used in genetic studies on MDD likely include cases who differ dramatically in both symptom profiles and severity. Depression is also heterogeneous in other aspects: patients differ in their onset of disease (e.g. adolescence versus old age), time course (single episode versus multiple episodes) and comorbidities—important dimensions that are often unmeasured and unmodeled in genetic studies. Variations in physiology at the cellular and molecular levels, such as tissue-specific gene expression and neuronal function, may present biological manifestations that underlie the above phenotypic differences.

Third, etiology, encompassing the diverse combination of *genetic, environmental and other* factors leading to one’s disease, as well as their interactions. Individuals may have different levels of genetic liability to depression through carrying different risk alleles at genetic loci with effects on the molecular pathways leading to the disease, and they may be exposed to different environmental factors that also add to their disease liability. Further, the effects on depression liability contributed by the risk alleles one carries may change depending on one’s physiological (through gene–gene interactions, GXG) and external environments (through gene–environment interactions, GXE).

Progress in the past years stems largely from genetics research studying heterogeneity in depression manifestations and etiology. Below, we review recent efforts to identify depression subtypes using clinical and data-driven approaches, examine differences in genetic architecture of depression across contexts, and discuss their promises and limitations. We argue that heterogeneity in operationalizations of depression cuts across these sections and is likely a considerable source of inconsistency.

## Using manifestations to understand etiology

Subtypes of depression have been proposed based on clinical observations and data-driven approaches, and research has largely focused on comparing their genetic architectures and how well they can be predicted with existing polygenic risk scores (PRS).

### Theory-driven depression subtypes

Decades of clinical experience and patients’ own accounts have led to clinical subtypes of MDD that are reflected in current DSM-5 specifiers such as atypical, melancholic and anxious depression. Subtypes have also been proposed based on developmental timing (19,20), treatment resistance (21) and recurrence (22). These clinical subtypes have been the primary target of genetic studies.

As an example of a symptom-based clinical subtype, atypical depression is primarily characterized by hypersomnia and weight gain, as opposed to depression more typically characterized by insomnia and weight loss. Typical and atypical depression subtypes differ in heritabilities (43% versus 38%, though with large standard errors), with PRS for other psychiatric traits showing stronger associations with the typical than atypical subtype (23). Conversely, PRS for immune-metabolic traits such as body mass index (BMI) and C-reactive protein are strongly associated with the atypical depression (24), and patients with the atypical subtype were found to carry more genetic risk variants for BMI and C-reactive protein (25). This suggests that atypical MDD may share greater genetic liability with immune-metabolic conditions (26).

In terms of developmental timing, genetic overlap between early and late-onset MDD has been shown to be only moderate (27). PRS from a recent GWAS meta-analysis of depression predicted early onset MDD is better than late-onset (5), and in hospital-treated cases the iPSYCH study, PRS from both bipolar disorder (BIP) and schizophrenia (SCZ) were associated with earlier MDD onset (28,29). Another longitudinal study found that PRS from SCZ and attention deficit hyperactive disorder (ADHD) were associated with early adolescent rather than later-adolescent onset trajectories, suggesting shared genetic contributions for early onset MDD and other psychiatric and neurodevelopmental conditions (30,31). Different heritabilities have also been found between depression occurring during the perinatal period (e.g. postpartum) and non-perinatal depression (44% versus 32%) (32), with preliminary evidence suggesting stronger associations between PRS of BIP and SCZ with perinatal depression than non-perinatal depression (33,34).

However, research into distinctions between subtypes, whether symptom- or timing-based, relies on data that is often not available. For example, The China, Oxford and Virginia Commonwealth University Experimental Research on Genetic Epidemiology (CONVERGE), due to its strict enrollment criteria, is the only genetically informed cohort with a high proportion of cases presenting with melancholic depression (35); no replication cohorts were available to date. Early GWAS attempts on other clinical features such as episodity (36) and treatment response (37–39) were limited in power and did not produce positive findings. As larger efforts have been recently invigorated (40,41), we may gain new insights with them.

### Data-driven depression subtypes

A body of complementary research has emerged to identify depression subtypes using agnostic, data-driven methods. There is over half a century of literature characterising depression heterogeneity based on symptom data. There are two principled ways, reviewed extensively elsewhere (42–45). First, exploratory and confirmatory factor analyses (exploratory factor analysis—EFA, confirmatory factor analysis—CFA) aim to identify underlying symptom dimensions using the symptom covariance matrix. Studies consistently extract more than two factors, and results largely depend on which symptoms are included (42,46–50). Second, latent class analysis (LCA) aims to determine more homogeneous subgroups of individuals. Across studies, the most consistent finding is that classes are often organized by severity on all symptoms (indicating a continuum rather than separate classes), though specific results are mixed and depend on assessment instruments (42,45,51,52). Overall, measurement heterogeneity across cohorts has made inferences challenging (42). Three further complications are that symptoms are often not fully assessed in controls due to skip-out assessments; analyses are often performed on cases using the very symptoms with which they were selected, incurring collider biases (53); and methods have assumptions, such as conditional independence in LCA, that are not always met (51,54).

Despite these challenges, there are increasing efforts to recover latent dimensions and classes at the genetic level. Building on the identification of three genetic factors reflecting mood, psychomotor/cognitive and neurovegetative features of MDD using twin modeling (55), a recent EFA on self-reported depression symptoms in UKBiobank obtained similar results and explored associations with depression PRS (56). A new framework, GenomicSEM, generalizes the structural equation modeling (SEM) approach to genetic covariance matrices (57), which can be generated from a joint analysis of GWAS summary statistics of individual depression symptoms, and can be used to test for genetic loadings on latent dimensions of depression.

Other data-driven approaches have been applied to physiological measures to identify etiologically meaningful subtypes. Variations of canonical correlation analysis (CCA) have characterized relationships between depressive symptoms and neuroimaging measures (58), and hierarchical clustering on resting-state fMRI measures have identified groups of depressed patients and their differential network dysfunctions (59), and machine learning methods have been used to cluster longitudinal responses to antidepressants to identify stable treatment response classes (60). In the future, these may be integrated with multi-omics for example, transcriptome-wide association (TWAS) approaches have begun to identify depression subtypes driven by brain and adipose tissue-specific gene expression (61).

Finally, genetic data have been used to directly identify data-driven subtypes. For example, subsets of MDD cases in UKBiobank with distinct genetic risks for SCZ, high neuroticism and early age of menopause (62) were identified using BUHMBOX (63), a statistical approach that involves identifying individuals who may carry genetic variants pleiotropic for other traits. Overall, continued efforts to incorporate new types of data and development of new data-driven methods hold great promise for subtype identification and validation.

## Contexts as part of etiology

This section reviews genetic investigations aiming to disentangle etiological heterogeneity across the contexts in which depression manifests. We also discuss challenges to these approaches, including measurement differences and ascertainment biases.

### Individual characteristics

Few genetic studies of depression have been performed in non-European populations, and the extent to which etiological factors for depression differ across populations remains unknown. A recent preprint compared ICD10-based MDD in individuals of African American ancestry (AA, *N* = 59 600) in the Million Veterans Program (MVP) in the USA with a meta-analysis of several depression cohorts of individuals with European ancestry (EUR, *N* = 1.1 million), including MVP (7). Although no GWAS hits for MDD were found in AA, likely due to insufficient power, 61% of the GWAS hits from depression in EUR showed the same directions of effect, suggesting a modest overlap in genetic factors leading to depression in people with both ancestries. This echoes results from a study comparing severe recurrent MDD of Han Chinese women (CHN, *N* = 10 640) in the CONVERGE cohort to MDD of EUR in various cohorts from the Psychiatric Genomics Consortium (PGC, EUR *N* = 18 662) (64). Low trans-ancestry genetic correlations were found between MDD in CHN and EUR (57–59) ($\rho$ = 0.33, 95% CI = 0.27–0.39), and the two GWAS hits from CONVERGE were not replicated due to drastic allele frequency differences (3,5,35).

In the studies discussed above, MDD from AA and CHN was compared with depression measured very differently in EUR. Despite reports of high rG between depression assessed in different ways within EUR to justify their use in cross-ancestry comparisons (rG = 0.81–1.07) (8,65), it has been demonstrated very clearly that they are distinct phenotypes with different genetic architectures (8). As such, genetic heterogeneity of depression may be overestimated across ancestries due to differences in operationalization. Differences in cultural norms around depression (15,16,66) and study participation (67) can incur ascertainment biases and further affect interpretation of results; assessing depression across populations requires greater efforts to understand how this condition manifests differently across settings.

This also applies to heterogeneity across other groups, including those defined by biological sex. Differences in MDD genetic architecture between sexes have been shown in both twin studies (68,69) and major GWAS cohorts (70), where heritability of MDD was found to be higher in females. However, this can be obfuscated by differences in operationalizations and ascertainment strategies between studies. Contrary to previous studies, heritability of depression in females was found to be lower in UKBiobank (8), and it is the only dataset whose PRS for depression in both sexes better predict MDD in males than females in an independent dataset (71). Ascertainment differences are likely to be a major contributor to this discrepancy, and minimizing such differences may unmask patterns across studies.

### Environments

Environmental factors contribute a large proportion of variability in depression risk, and stratifying depression cohorts by environmental factors may help identify differential genetic effects between those exposed and not exposed. For example, stratifying by exposure to stressful life events has revealed genetic heterogeneity in severe recurrent MDD from CONVERGE (72–74), with three significant GWAS hits and higher heritability of MDD in the non-exposed group (72), suggesting divergent genetic factors at play among the two groups. However, when MDD and stress exposure were differently defined in the UKBiobank, the opposite finding emerged, with higher heritability of MDD in the exposed group (75). Similar contradictions arose between two studies on interactions between MDD PRS and childhood trauma: in The Netherlands Study of Depression and Anxiety, MDD PRS was more strongly predictive of depression in trauma-exposed cases (76), while in RADIANT UK, it was more predictive of non-exposed cases (77). Further, a subsequent meta-analysis using cohorts ascertained with a range of strategies identified the third possible outcome—a null-finding (78). This non-replication was attributed to chance findings in the small cohorts used, and to a smaller extent gene–environmental (GE) correlation (78). But a further issue for replication may lie in operationalization differences between studies. Ascertainment biases, as well as heterogeneous measurement of both depression and stress exposures, may lead to differences in unmeasured environmental factors and inconsistencies in both polygenic and environmental contributions to disease liability. GXE effects detected between PRS and environmental contexts could therefore differ accordingly (78,79).

One potential solution is to target efforts at identifying and replicating GXE effects between single variants and environmental exposures. Though efforts to test single-variant GXE have often been thwarted by difficulties in correcting for confounding factors and a general lack of power, recently proposed methods may overcome this. StructLMM extends a linear mixed model approach to test random effects at genetic variants interacting with one or more environmental variables (80), and reverse GWAS (RGWAS) infers subtypes by clustering multiple traits and environmental factors, and tests for genetic heterogeneity between identified subtypes while robustly controlling for confounding factors (73). With larger datasets becoming increasingly available, these methods may start yielding results for depression.

## Way forward: splitting versus lumping

As depression may reflect several highly heterogeneous phenotypes, and it is difficult to agree upon a single construct that can be consistently measured, perhaps studying it at the level of a categorical diagnosis or dimensional symptom total score is not the only or best solution. Here, we discuss two alternative ways forward.

First, splitting, i.e. refocusing genetic discovery efforts on more granular phenotypes with higher validity and reliability such as individual symptoms (81,82). Recent studies have investigated genetic contributions to individual depressive symptoms (83–86) and how they vary across contexts (87). Analyses have shown that genetic contributions to individual symptoms are not equivalent to those for MDD (average rG = 0.6), nor to each other (rG range 0.6–0.9) (56,62). Going beyond symptoms, recent expansions in sequencing and phenotyping technologies such as neuroimaging (88–90) and molecular data (91,92) have enabled genetic analyses on endophenotypes (93). Though genetic contributions to such endophenotypes have not yet been found to have larger individual locus effect sizes (94) or to be any less polygenic (95) than complex diseases, they have been proposed to be more tractable (96). Clustering based on endophenotypes may reveal etiologically meaningful subtypes of depression, and investigating these may allow us to fill in the missing causal links between genetic variants and disease.

Second, lumping, i.e. moving beyond depression alone and embracing transdiagnostic features across related disorders. High comorbidity (97) and pleiotropy (98–100) between psychiatric disorders have motivated attempts to identify common genetic factors and implicated molecular pathways underlying multiple psychiatric disorders (57,101–103). Underlying liability for psychiatric conditions such as the *p* factor has been proposed (104), with preliminary evidence of a corresponding genetic basis (105).

Integrating both splitting and lumping, transdiagnostic insights gained from studies of endophenotypes may help us redefine diagnostic boundaries, a goal set out by the Research Domain Criteria (RDoC) 10 years ago (106).

## Conclusions

To summarize, we have identified three overarching sources of heterogeneity: operationalization, manifestations and etiology. The first pertains to heterogeneity in how we do science, the second and third to heterogeneity of the phenotype itself as well as its causes. Gaining a better understanding of how these three sources impact results in our field is a necessary (though not sufficient) step towards improving diagnostics and targeted treatments. From reviewing the literature, four lessons emerge.

(1) Measure consistently: Inconsistent findings in subtype identification and their genetic architecture are inevitable if depression is not operationalized consistently. Overall, this calls for harmonizing assessments of depression across studies.
(2) Measure more: Both theory- and data-driven approaches to disentangle the complex phenotype of depression rely on data, and even the most sophisticated statistical approaches cannot overcome missing input data. One crucial step forward is to assess a broader range of data—including individual depression symptoms and salient clinical characteristics such as age of onset, number of episodes and recurrence—and utilize them to study depression heterogeneity. Further, new types of data, including those from activity trackers in wearable technologies, text and voice through natural language processing, and longitudinal mood assessment by computerized adaptive screening questionnaires, may be helpful to identifying subtypes for genetic analysis.
(3) Collaborate: Complex traits like depression cannot be understood in a mono-disciplinary vacuum, because they require, in addition to knowledge of quantitative genetics, a nuanced understanding of the phenotype under investigation. The goal to identify and validate depression subtypes therefore calls for collaborations with patients, clinicians, epidemiologists, statisticians, computer scientists, sociologists, anthropologists and many others.
(4) Follow through: To make good on the promise of GWAS to deliver genetic insights that would improve diagnosis, treatment and prevention of depression in individuals with diverse etiological causes, we need to look beyond our findings of differences in genetic architecture and PRS associations. Fine-mapping using sequencing datasets may help identify candidate causal variants with heterogeneous effects on depression subtypes; integration of multi-omics data may point to the different tissues and biological pathways involved; single cell transcriptomics across developmental time points may lend spatial and temporal resolution; experimental designs in re-differentiated human induced pluripotent stem cells, organoids or model organisms may allow us to validate the biological relevance of effects we find and identify potential targets for drug interventions.

Doing all of this, we may find that depression really consists of an entangled web of partly overlapping biopsychosocial constructs, with overlapping genetic contributions and underlying biological mechanisms. Perhaps now is the right time for us to take the bold next step and acknowledge the complex reality that the field is searching for the genetic architecture of ‘depressions’ rather than depression. This is a challenge, but simultaneously a great opportunity and offers a clear path forward towards embracing the heterogeneity of depressions in our theories, measures and methods.

*Conflict of Interest statement.* The authors declare no conflicts of interest.

## Funding

National Institute of Mental Health (T32MH017119 to K.W.C.).
